# Ultrasonic Substrate Vibration-Assisted Drop Casting (SVADC) for the Fabrication of Photovoltaic Solar Cell Arrays and Thin-Film Devices

**DOI:** 10.1186/s11671-015-1168-9

**Published:** 2015-12-01

**Authors:** Morteza Eslamian, Fatemeh Zabihi

**Affiliations:** University of Michigan-Shanghai Jiao Tong University Joint Institute, Shanghai, 200240 China

**Keywords:** Solution-processed solar cells, Perovskite solar cells, Organic solar cells, Drop casting, Ultrasonic substrate vibration, Process scale-up

## Abstract

A simple, low-cost, versatile, and potentially scalable casting method is proposed for the fabrication of micro- and nano-thin films, herein termed as ultrasonic “substrate vibration-assisted drop casting” (SVADC). The impingement of a solution drop onto a substrate in a simple process called drop casting, usually results in spreading of the liquid solution and the formation of a non-uniform thin solid film after solvent evaporation. Our previous and current supporting results, as well as few similar reports by others, confirm that imposing ultrasonic vibration on the substrate can simply convert the uncontrollable drop casting method into a controllable coating technique. Therefore, the SVADC may be used to fabricate an array of emerging thin-film solar cells, such as polymer, perovskite, and quantum-dot solar cells, as well as other small thin-film devices, in a roll-to-roll and automated fabrication process. The preliminary results demonstrate a ten-fold increase in electrical conductivity of PEDOT: PSS made by SVADC compared with the film made by conventional drop casting. Also, simple planar perovskite solar cells made here using SVADC show promising performance with an efficiency of over 3 % for a simple structure without performing process optimization or using expensive materials and treatments.

## Background

The motivation behind the development of emerging photovoltaic thin-film solar cells, such as polymer, perovskite, and quantum-dot solar cells, is their potential to be fabricated using solution-processed molecular semiconductor materials and vacuum-free scalable casting methods. Among casting methods, spin coating is widely used, but its application is limited to lab-scale and batch processes. Dip coating and doctor blading are other lab-scale casting methods, which are less controllable. Organic solar modules have been fabricated in pilot scale using a combination of some of the scalable methods [1, 2]. A scalable casting technique should be ideally, fast, low-cost in terms of solar cell materials and energy consumption, roll-to-roll fabrication compatible, touch-free, automated, controllable, and suitable to coat small areas in an array of cells. The major potentially scalable casting techniques currently under investigation for the fabrication of emerging solar cells include the inkjet printing [3], slot-die casting [4], screen printing [5], gravure printing [6], spray coating [7, 8], etc. Each of the above-mentioned scalable techniques is usually suitable for the fabrication of films with particular characteristics. For instance, inkjet printing can direct small droplets to the target to make dots, lines and thin films. It is, however, a rather low-throughput process, given that the features are fabricated based on impingement and coalescence of many individual small droplets. Slot-die casting method is not suitable for the fabrication of nano-thin films, given that a rather large amount of solution is delivered during the deposition process, making the films thick. And while spray coating is a fast and scalable technique, it generally suffers from unsteadiness, inadequate film uniformity and intactness [8, 9]. Photovoltaic thin-film solar cells or modules are usually made of an array of small cells which are interconnected in series and parallel to achieve the required voltage and current [1, 2]. The size of each cell should be kept small to achieve a higher device performance and efficiency, given that large-area thin films are more susceptible to manufacturing defects and imperfections compared to small-area films. Therefore, masks may be required to be used during the spray deposition and other casting processes to ensure that only specific areas are covered, resulting in wastage of solar cell materials. To improve the uniformity of spray-on films, we have recently developed, implemented, and tested a modified coating technique, termed as substrate vibration-assisted spray-coating (SVASC), in which during the spraying process, controlled ultrasonic vibration is imposed on the substrate [10–12]. The imparted mechanical energy to the wet film leads to improvement in its uniformity as well its nano-structure and functionality. The imposed substrate vibration is also applicable to other casting methods, and has been used by others as well, such as for the fabrication of transistors [13] and nanowires [14]. It has been shown theoretically [15, 16] and experimentally [11, 12] that the imposed substrate vibration can improve or deteriorate surface wetting, depending on the amplitude, power and frequency of the imposed vibration. In the following sections, a manufacturing technique is proposed for the large scale fabrication of solar cell arrays that demonstrates continuous fabrication of solar cell layers on a solar cell module using drop casting combined with imposed substrate vibration, in a method herein termed as substrate vibration-assisted drop casting (SVADC).

## Presentation of the Hypothesis

Conventional drop casting is not considered as a film formation method, due to the lack of adequate control over the film characteristics. However, drop casting when combined with imposed ultrasonic substrate vibration can become controllable. In the proposed process, a small volume of a precursor solution with low surface tension is released from a capillary tube by dripping with negligible initial velocity or it is injected with an initial velocity and is directed toward the substratet. In either case, the drop impinges on the substrate with a velocity and momentum. The drop momentum results in substantial liquid spreading on the substrate. A wetting substrate with low interfacial tension and a high drop impinging velocity facilitate drop spreading. This is usually analyzed in terms of the Weber number (*We*), which is the ratio of the drop inertia force to the surface tension force. The drop Reynolds number (*Re*) also represents the ratio of the drop inertia to the viscous force. Therefore, the solution viscosity, density, surface tension, drop size, and impinging velocity via *Re* and *We* numbers, as well as surface wetting properties control drop spreading. If the surface is not sufficiently wetting and *Re* and *We* are not large enough, the wet film may retract and recede resulting in the formation of a non-uniform thin solid film after drying. An excessively large *Re* and *We* may cause drop splashing which is an unwanted phenomenon [8, 17]. To improve drop spreading and uniformity of the deposited film, the proposed SVADC method takes advantage of imposed ultrasonic substrate vibration with controlled vibration power (amplitude) and frequency. Another part of the novelty of this work is the way that the SVADC can be employed in an automated fabrication process, which is explained later.

## Testing the Hypothesis

**Fig. 1:**
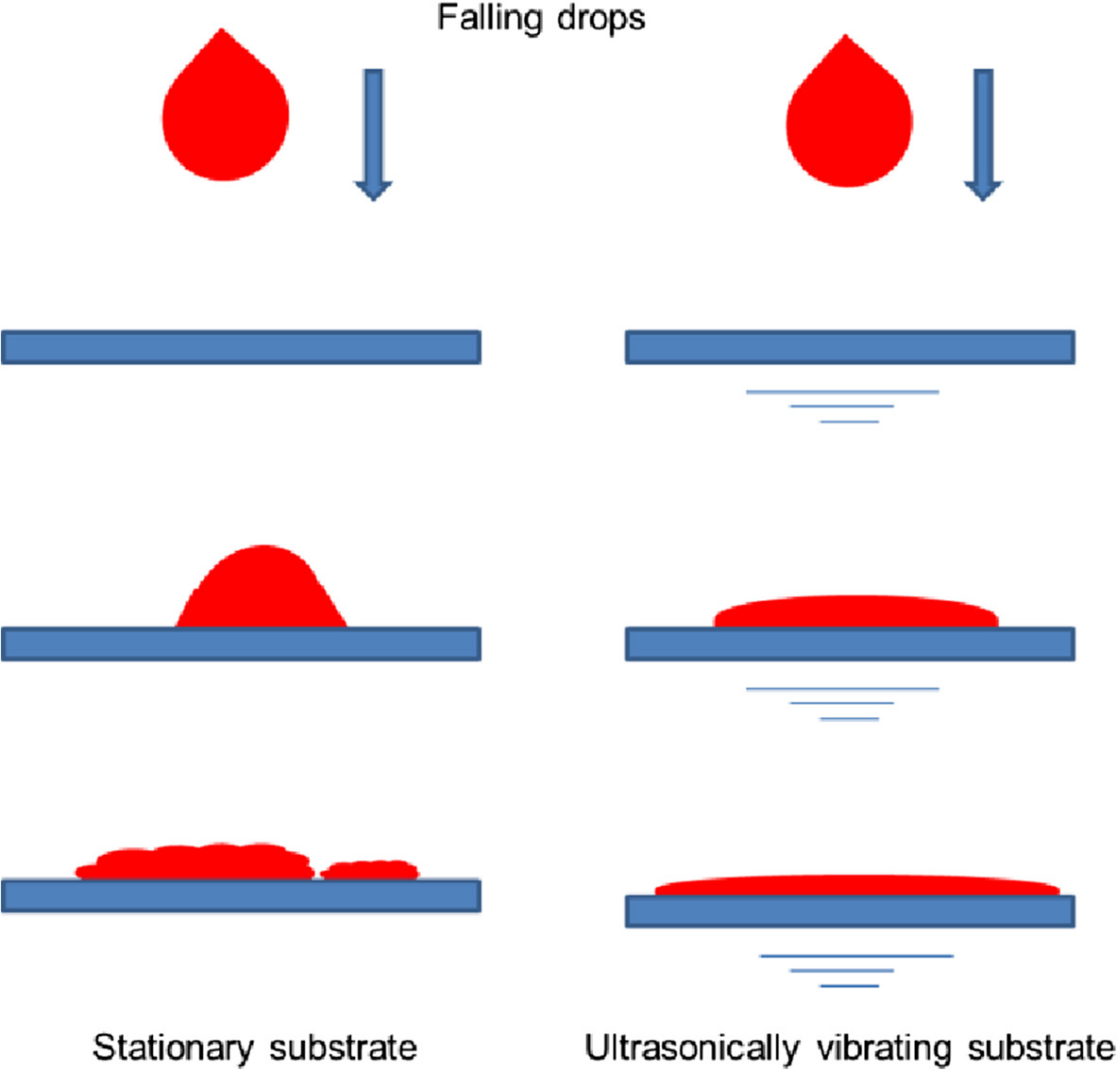
Drop impingement and spreading on a stationary substrate (left) and a vibrating substrate (right)

**Fig. 2:**
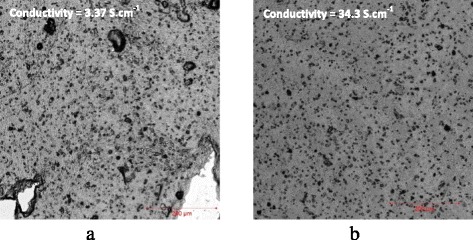
Laser images of the PEDOT: PSS films made by drop casting. **a** Case 2 of Table 1 (stationary substrate). **b** Case 6 of Table 1 (vibrating substrate). Electrical conductivities are shown on the images. The image area is 600 × 700 μm

Part of the hypothesis is backed by our previous studies on the effect of the imposed substrate vibration on topography and nano-structure of spray-on films. In one study, it has been observed that the uniformity, roughness, and electrical conductivity of spray-on PEDOT: PSS film significantly improves, if the substrate is subjected to a low-power ultrasonic vibration [11, 12]. Also, according to our unpublished data, the post treatment of wet spun-on PEDOT: PSS films by imposed substrate vibration results in up to twelve-fold increase in electrical conductivity, caused by an enhancement in the film internal and surface uniformity. The positive effect of the imposed substrate vibration on the film topography and the principle of operation of SVADC are schematically shown in Fig. 1. To demonstrate the effectiveness of the SVADC method, preliminary experiments were performed on 4-mm-sized free falling dripping drops of 1.3 wt.% PEDOT: PSS aqueous solution diluted with IPA at a volume ratio of 4:1, respectively, impinged on a smooth glass substrate from the above. In some cases, the glass substrate was placed atop a metal box attached to an ultrasonic transducer (Cheersonic Ultrasonic Co., China). The transducer was connected to a signal generator operating at a frequency of 40 kHz with variable power or vibration amplitude. More details may be found in Ref. [12]. The as-casted wet films were then dried and annealed for 25 min at 120 °C in an oven. Samples were analyzed using a confocal laser scanning microscope (CLSM; Zeiss, model LMS700, Germany). The CLSM-measured thickness and the area-averaged RMS roughness of some samples on an area of 600 × 700 μm are shown in Table 1. The results manifest that the application of imposed vibration results in significant reduction in film roughness and film thickness due to better drop spreading. Also, a larger free fall height (distance between the substrate and the initial position of the drop) results in lower roughness and thickness due to increased drop velocity and momentum at the time of impact. However, the free-fall height has nearly no effect if the power of the imposed ultrasonic vibration on the substrate is as high as 20 W, indicating that the substrate vibration is a strong factor controlling the film characteristics. The laser images of sample 2 (casted on stationary substrate with no vibration) and sample 6 (casted on vibrating substrate at 20 W) are shown in Fig. 2. It is observed that the imposed substrate vibration (Fig. 2b) results in the uniform distribution of black PEDOT: PSS grains within the film and reduced number of pinholes and imperfections. The rupture is clearly seen in the film made using conventional drop casting as shown in Fig. 2a. The electrical conductivity of the films, measured using the four-point probe method along a 6 mm straight line on the films [11] provided on the images of Fig. 2, shows an impressive ten-fold increase when ultrasonic vibration is imposed on the substrate (3.35 S cm^−1^ for the non-vibrating substrate versus 34.3 S cm^−1^ for the vibrating substrate). The slope of the I–V curves of the films, which are linear (not shown), and the film thickness were used to obtain the film electrical conductivity [11].

**Table 1:** Thickness and roughness of PEDOT: PSS films made by regular drop casting and substrate vibration-assisted drop casting; vibration time = 60 s

Case #	Vibration power (W)	Free fall height (cm)	Film thickness (μm)	Film roughness (μm)
1	0	2	18.2	8.98
2	0	10	10.3	5.35
3	5	2	2.93	2.09
4	5	10	2.41	1.86
5	20	2	1.80	0.90
6	20	10	2.00	0.86

Careful evaluation of the performance of the SVADC method, however, entails design of well-thought experiments, fundamental studies, and device fabrication and testing. Here just to demonstrate the merit of the SVADC method, a single planar mixed-halide perovskite solar cell was fabricated based on the simple planar FTO-coated glass/PEDOT: PSS/CH_3_NH_3_PbI_3 − *x*_Cl_*x*_/PCBM/Al architecture. The IPA solvent was added to PEDOT: PSS aqueous solution (1.3 wt.% PEDOT: PSS dispersed in water) with the volume ratio of 3:1, respectively. The PEDOT: PSS solution was dropped onto FTO-coated glass, forming a film which was annealed on a hot plate at 120 °C for 20 min. This is the hole-transporting layer of the cell. To prepare perovskite (active or light harvesting layer) reagents, MAI was dissolved in 2-propanol to obtain a solution with a concentration of 0.1 mg mL^−1^, and PbCl_2_ was dissolved in a 3:1 volume ratio of the mixture of DMSO and DMF solvents to obtain a solution with a concentration of 0.26 mg mL^−1^. PbCl_2_ solution was drop-casted atop PEDOT: PSS film, and the formed crystalline PbCl_2_ film was annealed at 90 °C for 30 min; then MAI solution was dropped atop the PbCl_2_ film. MAI solution reacts with PbCl_2_ film, making the mixed-halide perovskite CH_3_NH_3_PbI_3 − *x*_Cl_*x*_ film. The perovskite layer was annealed at 90 °C for 100 min. PCBM powder was dissolved in chlorobenzene forming a 50 mg mL^−1^ solution, and was dropped on the CH_3_NH_3_PbI_3 − *x*_Cl_*x*_ layer and annealed at 80 °C for 15 min. PCBM layer helps extraction and transport of electrons to the back contact. All layers were made by conventional drop casting, as well as SVADC in a glovebox at a vibration power of 5 W at 90 s, except the Al back contact, which was deposited by thermal evaporation. All chemicals were purchased from Sigma-Aldrich, USA.

**Fig. 3:**
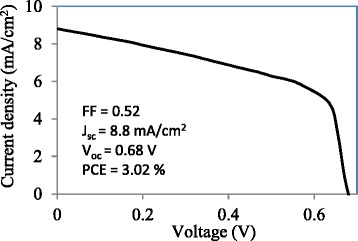
Current density-voltage curve of a planar FTO-coated glass/PEDOT: PSS/CH_3_NH_3_PbI_3 − *x*_Cl_*x*_/PCBM/Al perovskite solar cell made by substrate vibration-assisted drop casting (SVADC)

The current density-voltage curves and PCE of solar cell devices were obtained by a solar simulator and a source meter (National Instruments, model NI PXI-1033, TX, USA) under AM1.5G solar irradiation with intensity of 1000 W/m^2^. The cells made using regular drop casting showed no or only a weak output. Figure 3 shows the current density-voltage of a cell (2 × 2 mm) made by SVADC with a PCE of 3.02 % in a cell made with minimum process optimization and using a very simple but scalable process. This efficiency is lower than the efficiency of a somewhat similar device made by spin coating and spray coating (active layer only) [18]. In Ref. [18], the thickness of each layer was optimized and Ca/Al was used as the back contact instead of Al, to improve the energy band diagram and to facilitate charge collection. Therefore, performing process optimization on each layer and using Ca/Al as the back contact can further improve the short-circuit current density, open-circuit voltage, fill factor, and therefore the efficiency of the cell made by SVADC. Device optimization process was beyond the scope of this work.

## Implication of the Hypothesis

**Fig. 4:**
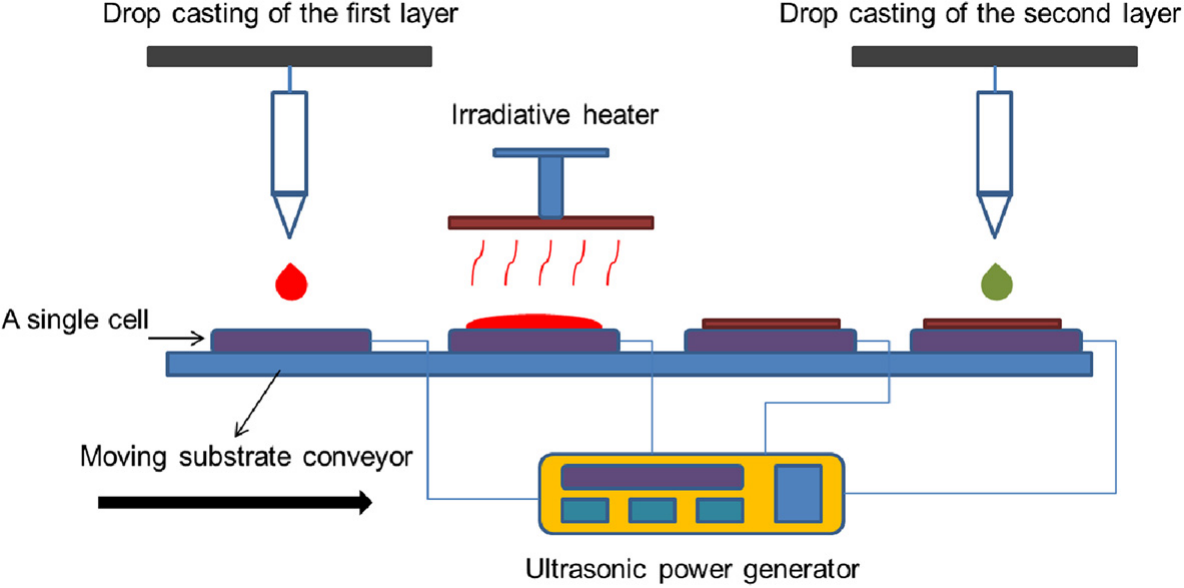
Schematic of proposed automated manufacturing apparatus incorporating SVADC for the fabrication and heat treatment of solar cell arrays

The proposed coating technique, SVADC, may be used for the fabrication of small-area coatings and thin-film devices in an automated process as well. One application is the photovoltaic solar cell arrays, made of several small cells fabricated on a panel moving by a conveyor as shown in Fig. 4. One or more nozzles or capillary tubes release one or few drops of a solar cell precursor solution onto small-area vibrating substrates to form various layers of thin-film solar cells, successively. The as-casted wet film may be exposed to an irradiative heater for film drying and post annealing, a process to enhance the film nano-structure. The process is roll-to-roll compatible if flexible substrates are used. Also, several layers of a thin-film solar cell may be deposited sequentially using the same manufacturing process. The maximum effective and uniform surface area that can be obtained for application in a device depends on the surface wettability, solution properties, impingement conditions, and substrate vibration.

## Abbreviations

CLSM: confocal laser scanning microscope
DMF: N,N-dimethylformamide
DMSO: dimethyl sulfoxide
FF: field factor
FTO: fluorine-doped tin oxide
IPA: isopropyl alcohol
J_sc_: short-circuit current density
MAI: CH_3_NH_3_I
PCE: power-conversion efficiency
PCBM: phenyl-C61-butyric acid methyl ester
PEDOT: PSS: poly(3,4-ethylenedioxythiophene)-poly(styrenesulfonate)
RMS: root-mean-square
SVADC: substrate vibration-assisted drop casting
V_oc_: open-circuit voltage
